# Deep proteome of human nNOS/NOS1-positive versus MOCK SH-SY5Y neuroblastoma cells under full nutrition, serum free starvation and rapamycin treatment

**DOI:** 10.1016/j.dib.2018.10.079

**Published:** 2018-10-26

**Authors:** Juliana Heidler, Lucie Valek, Ilka Wittig, Irmgard Tegeder

**Affiliations:** a Institute of Clinical Pharmacology, Goethe-University Hospital, Frankfurt, Germany; b Functional Proteomics, SFB815 Core Unit, Goethe-University, Frankfurt, Germany

## Abstract

Upregulations of neuronal nitric oxide synthase (nNOS/NOS1) in the mouse brain upon aging suggest a role in age-associated changes of protein homeostasis. We generated a cell model, in which constitutive expression of nNOS in SH-SY5Y cells at a level comparable to mouse brain replicates the aging phenotype i.e. slowing of cell proliferation, cell enlargement and expression of senescence markers. nNOS+ and MOCK cells were exposed to proteostasis stress by treatment with rapamycin or serum-free starvation. The proteomes were analyzed per SILAC or label-free using hybrid liquid chromatography/mass spectrometry (LC/MS). Full scan MS-data were acquired using Xcalibur, and raw mass spectra were analyzed using the proteomics software MaxQuant. The human reference proteome from uniprot was used as template to identify peptides and proteins and quantify protein expression. The DiB data file contains essential MaxQuant output tables and includes peptide and protein identification, accession numbers, protein and gene names, sequence coverage and quantification values of each sample. Differences in protein expression in MOCK versus nNOS+ SH-SY5Y cells and interpretation of results are presented in Valek et al. (2018). Raw mass spectra and MaxQuant output files have been deposited to the ProteomeXchange Consortium (Vizcaino et al., 2014) via the PRIDE partner repository with the dataset identifier PRIDE: PXD010538.

**Specifications table**

**Table:** 

Subject area	*Neuroscience, Proteomics*
More specific subject area	*Neurobiology, Aging, Protein homeostasis, Redox biology*
Type of data	*Spreadsheets*
How data was acquired	*Liquid chromatography / mass spectroscopy.* Thermo Scientific LTQ Orbitrap XL or Thermo Scientific™ Q Exactive Plus equipped with an ultra-high performance liquid chromatography unit and a Nanospray Flex Ion-Source, Xcalibur acquisition, MaxQuant search
Data format	Excel Table 1: MaxQuant data of normalized SILAC proteome (vehicle versus 6 h rapamycin)
	Excel Table 2: Gene Ontology analysis of significantly regulated proteins (SILAC experiment)
	Excel Table 3: MaxQuant data of label-free deep proteome (conditions: full medium, 24 h serum-free, 24 h rapamycin)
Experimental factors	*Cell treatment and harvest, protein extraction, storage at −80 °C, chromatography and MS analysis, MaxQuant software and Perseus software, gene ontology annotation*
Experimental features	*nNOS+ (neuronal nitric oxide synthase) and MOCK transduced SH-SY5Y neuroblastoma cells without and with rapamycin treatment for 6 h and 24 h or serum-free starvation for 24 h versus control conditions*
Data source location	*Frankfurt, Germany*
Data accessibility	*Data is with this article.*
	*Raw mass spectra and raw MaxQuant output tables, and a description of the sample processing are deposited to the ProteomeXchange Consortium**[2]**via the PRIDE partner repository with the dataset identifier* PRIDE: PXD010538, *which is publicly available*.

**Value of the data**

• The proteome data sets are useful to gain insight into NO-dependent and nutritient-dependent changes of protein homeostasis in the context of aging.
• The data sets provide information about the phenotypes of SH-SY5Y cells, which are the most widely used model in neurodegeneration research.
• The data may be used for comparison with proteome or transcriptome sets in models of aging or neurodegenerative diseases.

## 1 Data

We performed deep proteome analyses in MOCK and nNOS+ SH-SY5Y cells using SILAC and label free proteome analyses. Culture, transduction, stimulations and details about the data analysis are described in Ref. [1].

The data are MaxQuant output files (Table 1 SILAC, Table 3 LFQ proteome) including peptide and protein identification, accession numbers, protein and gene names, sequence coverage and SILAC or label free quantification (LFQ) values of each sample. Identifications from the reverse decoy database, identified by site only and known contaminants were excluded. The raw MS proteomics data of the Full Proteome have been deposited to the ProteomeXchange Consortium via the PRIDE [2] partner repository with the dataset identifier PXD010538. Project Webpage: http://www.ebi.ac.uk/pride/archive/projects/PXD010538

FTP Download: ftp://ftp.pride.ebi.ac.uk/pride/data/archive/2018/10/PXD010538

In addition, the Excel file contains a table (Table 2) with the Gene Ontology terms associated with the proteins, which accumulated in nNOS+ cells upon stimulation with rapamycin for 6 h in the SILAC experiment (raw data in Table 1). The GO overrepresentation analysis was performed with the DAVID Gene Functional Classification Tool (http://david.abcc.ncifcrf.gov) [3], [4].

## 2 Experimental design, materials and methods

### 2.1 Cell culture and transduction

Briefly, SH-SY5Y were grown in RPMI 1640 medium supplemented with heat-inactivated 10% fetal bovine serum, 100 U/ml penicillin/streptomycin, and 2 mM glutamine at 37 °C, 5% CO_2_ in humidified atmosphere. For SILAC experiments (stable isotope labeling by amino acids in cell culture), cells were grown in SILAC™ Protein ID & Quantitation Medium (Invitrogen) containing SILAC stable isotopic [^13^C_6_]-L-arginine and [^13^C_6_] L-lysine and supplements as above for at least 10 passages.

A stable cell line of nNOS expressing SH-SY5Y cells was produced by lentiviral-mediated transduction of nitric oxide synthase 1 (nNOS/NOS1) using a bicistronic NOS1-IRES-EGFP expression vector (GeneCopoeia, Mm04153 pReceiver-Lv; IRES, internal ribosomal entry site). Control cells were transduced with the control lentiviral vector and are referred to as MOCK. Transduced cells were FACS sorted according to their GFP expression (FACS-Aria Cell sorter, Becton Dickinson).

To induce mTOR-dependent autophagy MOCK and nNOS+ cell were grown to 60–70% confluence and were stimulated with 1 µM rapamycin for 6 h or 24 h. An equal volume of vehicle was added to the control cells. During stimulation, cells were supplemented with NOS cofactors including 10 µM NAD, 40 µM NADPH, and 100 µM tetrahydrobiopterin. For starvation, cells were washed, and cultured in serum-free medium for 24 h.

### 2.2 Super SILAC analysis of protein abundance and SNO-modifications

The SNO-Super SILAC method employs standards of cells grown in SILAC medium supplemented with peptides to detect SNO-sites. The standards were split in 2 parts, one labeled with iodoacetamide (IAM) and the other with N-ethylmaleimide (NEM). The method allows for simultaneous quantification of protein expression and S-nitrosylation.

Cell pellets of cells grown in SILAC medium (vehicle treatment) or control medium (rapamycin) were homogenized in Lysis buffer (50 mM Tris, 150 mM NaCl pH 7.6, 4% SDS, protease inhibitors (Roche)) containing 25 mM NEM and neocuproine, and incubated for 5 min at room temperature to block free thiols of cysteines. Protein extracts of cells grown in SILAC medium were split into two parts, one for labelling with 40 mM iodoacetamide (IAM) and the other with 40 mM N-ethylmaleimide (NEM). After centrifugation (16,000 *g*, 20 min, room temperature) the solubilized proteins were precipitated in ice-cold acetone overnight at −20 °C and then dissolved in Lysis buffer. Protein concentrations were measured with a GE NanoVue Spectrophotometer. SILAC experiments were done in triplicates.

For LC-MS/MS analysis, 20 µg of protein sample were added to 200 µl UA buffer (8 M urea in 0.1 M Tris/HCl pH 8.5) and transferred to a spin filter (microcon-30, Millipore) for further reduction, alkylation and digestion similar to Filter Aided Sample Preparation (FASP) described in Ref. [5]). The mixture was centrifuged at 10,000 rpm for 15 min at room temperature. S-nitrosylations in the sample were then reduced in 200 µl ASC/IAM solution (1 mM ascorbate, 50 mM IAM, 1 µM CuSO_4_) for 45 min followed by addition of 20 µg Super SILAC standard [6] supplemented with peptides to identify SNO-sites, and centrifugation (14,000 *g* for 40 min, room temperature). Samples were washed in UA buffer and again centrifuged at 14,000 *g* for 40 min. Oxidized thiols (disulfides and reversible oxidation) were reduced in 10 mM TCEP in 200 µl UA buffer for 10 min and alkylated by addition of 40 mM NEM and incubated for another 30 min. Samples were finally washed twice with 100 µl of UB buffer (8 M Urea, 100 mM Tris pH 8) and twice with 100 mM ammonium bicarbonate (ABC). The filtrate was harvested after each filtration step. 150 µl ABC (ammonium bicarbonate) containing 400 ng trypsin (1:100 ratio to protein) were added, mixed for one minute and then incubated overnight at 37 °C in a wet chamber. The peptides were collected after centrifugation in the filtrate. FASP filters were washed with 50 µl 0.5 M NaCl and the filtrate pooled and acidified with TFA (final concentration 0.1%). Samples were desalted on SPE columns (MILLI-SPE Extraction disk cartridge (C18-SD) and eluted in 70% acetonitrile, 0.1% TFA (CF3COOH) and dried in a SpeedVac™ (Thermo Scientific). To reduce the complexity, peptides were separated offline on a SCX column and 12 fractions were collected by a micro fraction collector (Sun Chrom) [7].

### 2.3 Mass spectrometry and peptide/protein identification for SILAC

Trypsinized peptides were dissolved in 5% acetonitrile supplied with 0.5% formic acid in water and separated on a 75 μm ID emitter tip (NewObjectives) filled with ReproSil-Pur C18-AQ 120 °A, 3 µm (Dr. Maisch GmbH) and placed into the autosampler of the liquid chromatography unit (Agilent 1200 Nano-HPLC). Nano-HPLC runs (90 min for SILAC) were performed with an increasing acetonitrile (ACN) gradient from 5% to 50% containing 0.1% formic acid with a flow rate of 200 nl/min. Subsequently, the column was washed with 90% ACN for wash-out and re-equilibrated with 5% ACN, 0.1% formic acid. Eluted peptides were automatically submitted to ESI-MS/MS measurements by LTQ Orbitrap XL™ ETD (Thermo Scientific).

The MS data were acquired by a survey scan in a mass range of 400 to 1600 *m*/*z* followed by CID fragmentation of up to 5 precursor ions using Data Dependent Acquisition (exclusion time 3 min, rejection of singly charged precursor ions). SILAC raw data were analyzed by MaxQuant (1.3.0.5) [8], [9] using the human uniprot database as template (August 2014, 68379 sequences). The MaxQuant settings were as follows: maximum missed cleavages 2, main search precursor mass tolerance 5 ppm, fragment ion tolerance 0.5 Da, optional modifications allowed on methionine (oxidation) and cysteine (modification by NEM and carbamidomethylation by iodoacetamide) and acetylation at the N-terminus of proteins. FDR was set to 0.05. Known contaminants and reverse hits were excluded from the protein list. SILAC Light/Heavy pairs excluding carbamidomethylated and NEM labeled peptides were used for protein quantification (DIB Table 1). Sequence coverage and peptide spectra were analyzed by PEAKS7 (Bioinformatics Solutions Inc, Waterloo, Canada).

### 2.4 Sample preparation for label-free full proteome analysis

After 24 h culture in full medium, 24 h starvation or rapamycin treatment, cells were washed twice with PBS, scraped off and pelleted by centrifugation. Cell pellets were homogenized in 1% Triton, 100 mM Tris pH 7.4 using a motor-driven Potter–Elvehjemn with 15 strokes and incubated on ice for 15 min. Following centrifugation at 16,000 rpm for 10 min at 4 °C, the supernatant was transferred to a new tube and precipitated with 20% TCA. The pellet was resolved in extraction buffer (10% SDS, 150 mM NaCl, 50 mM HEPES pH 7.8). Precipitated proteins were washed twice with ice-cold acetone and finally resolved in extraction buffer. Sonification for 5 s facilitated resolubilization of proteins. 100 µg of protein from each fraction was diluted in 4% (w/v) SDS, 100 mM HEPES, pH 7.6, 150 mM NaCl, 0.1 M DTT, mixed with 200 µl 8 M Urea, 50 mM Tris/HCl, pH 8.5 and loaded onto spin filters with a 30 kDa cut off (Microcon). The filter aided universal sample preparation protocol (FASP) [5] was used as described. Proteins were digested overnight with trypsin/LysC (sequencing grade, Promega). Following the protocol of Kulak and Mann [10], acidified peptides (final concentration 0.1% v/v TCA) were fractionated on multi-stop-and-go tips (StageTips) composed of C18-tips and strong cation exchange (SCX) tips. Peptides from the pellet fraction were eluted in three steps. Peptides from the supernatant fraction were eluted in six steps. All fractions of each sample were eluted in wells of microtiter plates. Peptides were dried and resolved in 1% acetonitrile, 0.1% formic acid.

### 2.5 Label free mass spectrometry and peptide/protein identification

For label-free deep proteome analysis, the LC/MS was performed on a Thermo Scientific™ Q Exactive Plus equipped with an ultra-high performance liquid chromatography unit (Thermo Scientific Dionex Ultimate 3000) and a Nanospray Flex Ion-Source (Thermo Scientific). Peptides were loaded on a C18 reversed-phase precolumn (Thermo Scientific) followed by separation on in-house packed picotip emitter tips (diameter 100 µm, 15 cm long from New Objectives) with 2.4 µm Reprosil C18 resin (Dr. Maisch GmbH). The gradient for full proteome analysis was from mobile phase A (4% acetonitrile, 0.1% formic acid) to 80% mobile phase B (80% acetonitrile, 0.1% formic acid) for 90 min with a flow rate 400 nl/min. MS data were recorded by data dependent acquisition Top10 method selecting the most abundant precursor ions in positive mode for HCD fragmentation. Lock mass option [11] was enabled to ensure high mass accuracy between multiple runs. The full MS scan range was 300–2000 *m*/*z* with resolution of 70,000 and an automatic gain control (AGC) value of 3*10^6^ total ion counts. The maximal ion injection time was 160 ms. Higher charged ions (2+) were selected for MS/MS scans with a resolution of 17,500, an isolation window of 2 *m*/*z* and an automatic gain control value set to 10^5^ ions. The maximal ion injection time was 150 ms. Ions were excluded if they occurred within a time-window of 20 s following a fragmentation event. Full scan data were acquired in profile mode and fragments in centroid mode by Xcalibur software.

Xcalibur raw files were analyzed using the proteomics software MaxQuant (1.5.2.8) [8] to identify peptides and proteins using the human uniprot reference proteome as template (Download June 2015, 76,086 entries). The false discovery rate (FDR) was set to 1%. The data file includes peptide and protein identification, accession numbers, protein and gene names, sequence coverage and label free quantification (LFQ) values of each sample. Identifications from the reverse decoy database, identified by site only and known contaminants were excluded.

MaxQuant data were further analyzed with ArrayStar (DNASTAR 15) and Perseus 1.6.0.2. [12] as described in the main manuscript [1].

## References

[1] Valek L., Heidler J., Scheving R., Wittig I., Tegder I. (2018). Nitric oxide contributes to protein homeostasis by S-nitrosylations of the chaperone HSPA8 and the ubiquitin ligase UBE2D. Redox Biol..

[2] Vizcaino J.A., Deutsch E.W., Wang R., Csordas A., Reisinger F., Rios D., Dianes J.A., Sun Z., Farrah T., Bandeira N., Binz P.A., Xenarios I., Eisenacher M., Mayer G., Gatto L., Campos A., Chalkley R.J., Kraus H.J., Albar J.P., Martinez-Bartolome S., Apweiler R., Omenn G.S., Martens L., Jones A.R., Hermjakob H. (2014). ProteomeXchange provides globally coordinated proteomics data submission and dissemination. Nat. Biotechnol..

[3] Dennis G., Sherman B.T., Hosack D.A., Yang J., Gao W., Lane H.C., Lempicki R.A. (2003). DAVID: database for Annotation, Visualization, and Integrated Discovery. Genome Biol..

[4] Huang D.W., Sherman B.T., Tan Q., Collins J.R., Alvord W.G., Roayaei J., Stephens R., Baseler M.W., Lane H.C., Lempicki R.A. (2007). The DAVID gene functional classification tool: a novel biological module-centric algorithm to functionally analyze large gene lists. Genome Biol..

[5] Wisniewski J.R., Zougman A., Nagaraj N., Mann M. (2009). Universal sample preparation method for proteome analysis. Nat. Methods.

[6] Deeb S.J., D׳Souza R.C., Cox J., Schmidt-Supprian M., Mann M. (2012). Super-SILAC allows classification of diffuse large B-cell lymphoma subtypes by their protein expression profiles. Mol. Cell Proteom..

[7] Washburn M.P., Wolters D., Yates J.R. (2001). Large-scale analysis of the yeast proteome by multidimensional protein identification technology. Nat. Biotechnol..

[8] Cox J., Mann M. (2008). MaxQuant enables high peptide identification rates, individualized p.p.b.-range mass accuracies and proteome-wide protein quantification. Nat. Biotechnol..

[9] Tyanova S., Mann M., Cox J. (2014). MaxQuant for in-depth analysis of large SILAC datasets. Methods Mol. Biol..

[10] Kulak N.A., Pichler G., Paron I., Nagaraj N., Mann M. (2014). Minimal, encapsulated proteomic-sample processing applied to copy-number estimation in eukaryotic cells. Nat. Methods.

[11] Olsen J.V., de Godoy L.M., Li G., Macek B., Mortensen P., Pesch R., Makarov A., Lange O., Horning S., Mann M. (2005). Parts per million mass accuracy on an Orbitrap mass spectrometer via lock mass injection into a C-trap. Mol. Cell Proteom..

[12] Cox J., Mann M. (2012). 1D and 2D annotation enrichment: a statistical method integrating quantitative proteomics with complementary high-throughput data. BMC Bioinform..

